# Pathological complete response to neoadjuvant chemotherapy, but not the addition of carboplatin, is associated with improved survival in Chilean triple negative breast cancer patients: a report of real world data

**DOI:** 10.3332/ecancer.2021.1178

**Published:** 2021-02-01

**Authors:** Benjamín Walbaum, Francisco Acevedo, Lidia Medina, M Loreto Bravo, Tomas Merino, Mauricio Camus, Francisco Dominguez, Sebastián Mondaca, Héctor Galindo, Bruno Nervi, Carolina Ibañez, Jorge Madrid, Sabrina Muñiz, José Peña, Érica Koch, Marcelo Garrido, Mauricio P Pinto, César Sánchez

**Affiliations:** 1 Department of Hematology-Oncology, Faculty of Medicine, Pontificia Universidad Católica de Chile, Diagonal Paraguay 362, Santiago, Chile; 2 Cancer Center ‘Nuestra Señora de la Esperanza’, Red de Salud UC Christus, Pontificia Universidad Católica de Chile, Santiago, Chile; 3 Department of Surgical Oncology, Faculty of Medicine, Pontificia Universidad Católica de Chile, Santiago, Chile; 4 Complejo Asistencial Hospital Dr Sotero del Rio, Santiago, Chile

**Keywords:** breast neoplasm, triple negative, neoadjuvant, chemotherapy, carboplatin

## Abstract

**Background:**

Breast cancer (BC) is the leading cause of cancer death for Chilean women. About 11% of cases are triple-negative (TN) BC. These are characterised by poor prognosis, higher risk of early recurrence and visceral dissemination versus other BC subtypes. Current standard treatment for early-stage non-metastatic TNBC patients consists of neoadjuvant chemotherapy (NACT) followed by surgery and radiotherapy. Pathological complete response (pCR) to NACT is associated with an increase in survival rates. In general, NACT and adjuvant regimens involve similar cytotoxic drugs. Recent studies have postulated that the use of platinum compounds in TNBC would increase response rates. However, their effects on patient survival remain uncertain.

**Materials and methods:**

We retrieved and analysed medical records from a total of 156 Chilean stage I–III TNBC female patients that received NACT and compared survival rates using carboplatin (Cb)-containing versus non-Cb-containing regimens at two health cancer centres.

**Results:**

Median age was 51 years (range: 24–81); 13.5% (*n* = 21) received Cb-containing regimens, 80.1% (*n* = 125) received sequential anthracyclines plus taxanes; 29.5% (*n *= 46) of the total group achieved pCR, 28% for the standard treatment and 35% (*n* = 8) for the Cb-containing group (*p* = 0.59). We confirmed pCR was associated with prolonged overall survival, invasive and distant disease-free survival (Log-rank *p* = 0.0236). But the addition of Cb was not associated with differences in survival measures (Log-rank *p* = 0.5216).

**Conclusions:**

To the best of authors’ knowledge, this is the first report on real-world data in the Chilean population assessing the effect of Cb-containing NACT in TNBC. The authors’ results suggest no survival benefit by the addition of Cb to standard NACT. However, we confirm an increase in survival associated to pCR regardless of treatment.

## Background

Breast cancer (BC) is the leading cause of cancer death for Chilean women. Only in 2018, an estimated total of 5,400 women were diagnosed with BC causing 1,700 deaths [1]. Furthermore, recent reports in Western countries (including Chile) have revealed an increase in BC incidence [2–4]. These could be explained by a combination of factors such as changes in lifestyles, mainly associated with an ageing population, increased exposure to hormones and obesity rates along with better diagnostic methods.

Within this context, triple negative breast cancers (TNBCs) represent about 11% of cases in Chile. This subtype is characterised by its poor prognosis and higher recurrence rates versus other subtypes [5]. Current standard of care for stage I–III TNBC involves neoadjuvant chemotherapy (NACT) followed by surgical tumour removal. Indeed, NACT improves breast conservation levels in TNBC [6] plus *in vivo* assessment of therapy response. Nonetheless, these regimens have failed to improve survival compared to adjuvant chemotherapy (CT) [6]. Despite this, studies demonstrate that a post-NACT pathological complete response (pCR) is a key prognostic marker that defines post-surgery therapeutic strategies [7]. CT regimens usually include alkylating agents, taxanes and anthracyclines that achieve ~40% of pCR [7]; the addition of platinum compounds increases these percentages by 1.5 or 2 fold. Therefore, recent studies have proposed its addition to regular NACT schemes, however, given the added toxicity and the uncertain overall survival (OS) benefit, this is still controversial in management guidelines [8–10].

To date, the effect of platinum compounds on pCR or OS rates in Chilean TNBC patients remains unreported. Moreover, its usage is not incorporated into national management guidelines. Herein, we assessed pCR and survival rates in localised TNBC patients that received platinum-containing NACT at two cancer centres in the Santiago metropolitan area.

## Materials and methods

### Study design and ethics approval

This was a retrospective study conducted at two cancer centres: the Pontificia Universidad Católica de Chile and Dr. Sótero del Río hospitals. The Human Research Ethics Committee approved this study (approval number: #200303006). All patient procedures strictly adhered to the principles of the Declaration of Helsinki (updated in 2013) and protected patient privacy according to ethical/legal standards.

### Patients and clinical data

Clinical and epidemiological data were obtained from medical records. The authors’ study included stage I–III TNBC patients selected for NACT at the Pontificia Universidad Catolica de Chile Cancer Center or the Dr. Sótero del Río hospital over the period 1997–2019. Hormone receptor status was inferred from pathology reports; TNBC was defined by ASCO-CAP criteria [11] as tumours that displayed <1% positivity on nuclear staining for oestrogen or progesterone receptors in tumour cells and an immunoscore of 0 or 1+ for human epidermal growth factor-type 2 receptor (HER2) or 2+ in the absence of amplification by fluorescent in situ hybridization (FISH). Clinical characteristics included NACT-platinum use, pCR rates defined as the absence of residual invasive disease on evaluation of the resected breast and lymph nodes after completing NACT (ypT0 ypN0 based on the American Joint Committee on Cancer staging system [12]).

### Statistical analysis

Patients’ OS were calculated from the date of diagnosis until the date of death or last follow-up (censored: August 2019). Similarly, Invasive Disease Free Survival (IDFS) was defined as the time from diagnosis until recurrence of invasive BC; Distant Disease Free Survival (DDFS) was defined as the time from diagnosis until systemic recurrence of BC. For the analysis of categorical variables, we used the chi-square test. The effects of systemic therapy regimens upon survival were assessed using the Kaplan–Meier method and the Log Rank test for equality of survivor functions. In cases where hazards were non-proportional (crossed survival curves), the Wilcoxon test was applied. Statistical significance was set at *p* < 0.05. All data were analysed using STATA software version 16.1 (StataCorp LP, College Station, Texas, USA).

## Results

A total of 156 stage I–III TNBC patients were analysed. Their main characteristics are summarised in Table 1. Median age at diagnosis was 51-year-old (range: 24–81) and the initial TNM stage distribution was 3%, 44% and 52% for I, II and III; respectively. Six patients (3.8%) had *BRCA* 1/2 mutations, 80.7% received anthracycline plus taxane schemes and 13% had carboplatin (Cb) added to their regimens. Patients that received Cb-containing NACT were predominantly from the private centre (66%) and consequently Cb was more frequently used in this group compared to women at the public hospital (28% versus 8%; *p* = 0.046).

Patients in the Cb group tended to be younger versus non-Cb counterparts; however, this difference did not reach statistical significance (49-year-old versus 52-year-old, respectively; *p* = 0.34). Additionally, no age differences were found for clinical stage, pCR, death or recurrence rates. On average, 29% of the total of patients achieved pCR; 28% with the standard treatment and 35% in the group that received Cb-containing NACT (*p* = 0.59, Table 1). Patients that did not receive sequential anthracyclines plus taxanes regimens (19.7%) still received either anthracyclines or taxanes (75%, *n* = 24 and 22% *n* = 7; respectively) but displayed lower pCR rates (31.7% versus 19%; *p* = 0.17). When Cb use was excluded, this difference was even more evident (32% versus 13.7%; *p* = 0.052).

**Figure 1.**
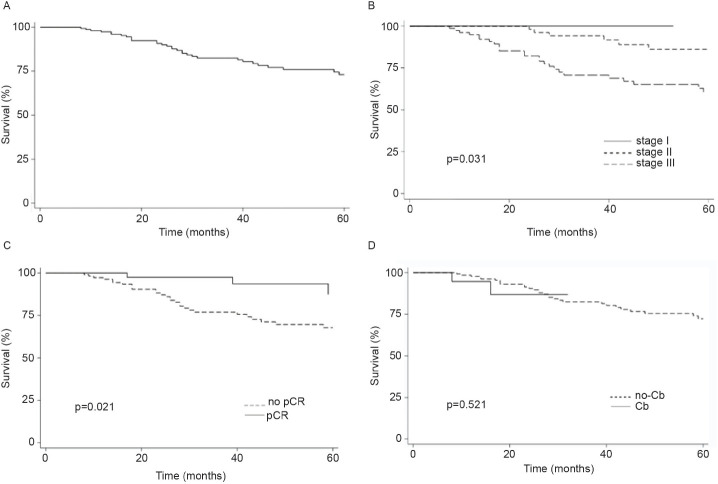
(a): OS in TNBC patients, (b): by clinical stage, (c): presence/absence of pCR or (d): carboplatin (Cb) use.

On the other hand, a lower TNM BC stage was associated with an increase in pCR rates (40% for stage II versus 19% for stage III; *p* = 0.012). At a 39-month median follow up, 22% of patients were deceased (35/156) and 28% relapsed with invasive disease (43/154). Median OS was not reached for the entire analysed group (Figure 1a). As expected, stage III patients displayed a lower OS versus stages I or II (Log-rank test *p* = 0.0028, Figure 1b). Next, we analysed survival rates in patients that displayed pCR. Indeed, these patients had a longer OS compared to patients without pCR (Log-rank *p* = 0.0236, Figure 1c).

**Figure 2.**
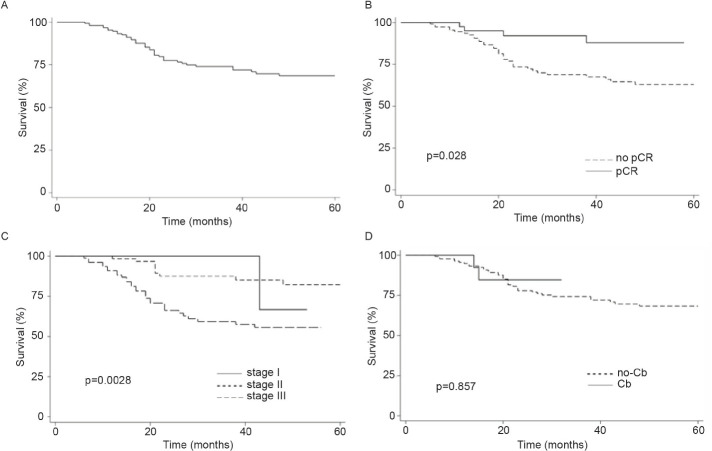
(a): IDFS in TNBC patients (b): by clinical stage, (c): presence/absence of pCR or (d): carboplatin (Cb) use.

**Figure 3.**
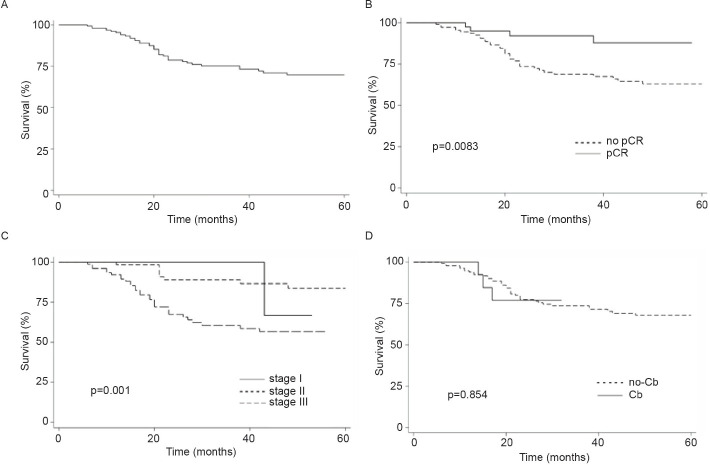
(a): DDFS in TNBC patients (b): by clinical stage, (c): presence/absence of pCR or (d) carboplatin (Cb) use.

Finally, the addition of Cb to NACT did not modify OS (Log-rank *p* = 0.5216, Figure 1d). In contrast, pCR and stage were associated with prolonged IDFS (Figure 2a–c) and DDFS (Figure 3a–c); whereas the addition of Cb had no significant impact in IDFS or DDFS (Figures 2d and 3d).

## Discussion

Every year, an estimated 5,500 new cases of BC are diagnosed in Chile [1]. From these, approximately 600 correspond to TNBC. Within this subset, the vast majority (92%) are early stage cases [5]. In 2016, the Chilean a BC Ministry of Health implemented the GES (Explicit Guarantees in Health) programme that guarantees the access and opportunity to receive treatment to patients with suspected and/or diagnosed BC. However, GES does not include Cb for localised TNBC [13]. The authors’ study provides real world evidence, seeking to contribute in the discussion of adding Cb as part of the standard neoadjuvant treatment for TNBC patients.

The authors’ findings indicate a significant relationship between pCR and survival, confirming previous reports in TNBC [7]. Although the addition of Cb was associated with a discrete increase in pCR, it was not associated with better survival. This could be explained by a combination of relatively small numbers of patients and low levels of pCR in the authors’ cohort. Also, the addition of platinum in a subset of patients could have been part of a ‘salvaging’ strategy for those with a poor initial response to conventional therapies (without platinum compounds). Thus, obviously these patients had worse prognosis. Still, patients who received anthracycline plus taxane regimens displayed a clear trend towards increased pCR compared to first or second generation regimens, with or without Cb [14].

A number of prospective studies have evaluated the role of platinum based NACT in TNBC (shown in Table 2). Firstly, the Spanish group GEICAM [15] compared the effect of Doxorubicin plus Cyclophosphamide and Paclitaxel (ACT) with or without Cb in TNBC and found no differences. This study defined TNBC by cytokeratin 5/6+ IHC and/or HER1-positivity; pCR (ypT0N0) was 30% in both groups. Second, the CALGB 40603/Alliance [16] study randomised patients undergoing paclitaxel-based CT into two arms, with or without Cb followed by AC. This study found a significant benefit in pCR for the platinum group (54% versus 41%). In line with the authors’ findings, pCR was associated with better OS and DFS regardless of treatment [17]. Third, the GeparSixto trial [18] randomised patients treated with paclitaxel plus liposomal doxorubicin and bevacizumab into placebo or Cb arms. In this case, pCR rates were 37% versus 53.2% (*p* = 0.005), respectively. Forty-seven month follow-up [16, 19] showed a 10% benefit in 3-year DFS (HR 0.56, 95% CI: 0.34–0.93; *p* = 0.02) for the platinum group, but no benefit in OS. Importantly, the initial design of this trial was not powered for survival analysis and no alkylating drugs were included in either group. This study also included a subgroup analysis by *BRCA* mutational status and Homologous Recombination Deficiency (HRD) as biomarkers of platinum-response, confirming only HRD as a predictor of response. Finally, the BrighTNess was a multicenter randomised phase III study [20] that compared three NACT arms: T-AC (Paclitaxel weekly for 12 followed by 4 cycles of doxorubicin and cyclophosphamide), plus the combination of veliparib (a poly adenosine ribose polymerase inhibitor (PARPi)) and Cb (TVCb-AC) or Cb only (TCb-AC). Patients were stratified by BRCA mutational status. This study confirmed a pCR benefit given by Cb: 58% in the TCb-AC group, 31% in the T-AC group and 53% with TVCb-AC (*p* < 0.001). Consequently no benefit was observed by the use of PARPi, nor with Cb in *BRCA* mutant or wild type patients.

All studies demonstrated a significant increase in grade 3/4 toxicities by platinum compounds, mainly due to myelosuppression and neutropenia (56%) and thrombocytopenia in 12%–20% of patients [16, 21], which leads to dose reduction in 68% of cases, and discontinuation in 48% [18]. In summary, phase 2/3 and meta-analysis studies [9, 10] demonstrate that the addition of platinum compounds in NACT increases pCR rates in TNBC [22]. More recently, a meta-analysis [23] reported a significant OS benefit in various neoadjuvant-platinum trials.

Still, this lack of consistency in survival benefits for platinum based regimes could be attributed to several reasons. First, as explained above, this could be due to a lack of statistical power derived from small patient samples. Second, because of patient selection bias; where the addition of Cb in patients with initial good prognosis will not modify significantly survival rates. Third, obtaining pCR in some patients could be related to other factors (i.e., not caused by therapeutic interventions) such as increased lymphocytic infiltration [24] that determine a better overall prognosis, regardless of CT regimens. Therefore, pCR would be an intrinsic marker of good prognosis. Fourth, in some patients the increase of pCR could be at the expense of reducing minimal residual disease post-NACT. And minimal residual disease, at least theoretically, could have an equivalent prognosis to those with a ypT0N0 [25]. Thus, an increase in pCR in these cases would not be reflected in increased survival. Finally, the CREATE-X study [26] showed benefits in prolonging DFS and OS with adjuvant therapy in TNBC patients with residual disease, blurring an accurate assessment of the effect of pCR or Cb in NACT. Even more, recently published data from the KEYNOTE522 trial [27] demonstrated increases in pCR and DFS by neoadjuvant pembrolizumab in patients that received Cb.

This work has several limitations, mainly derived from its retrospective nature. First, only a small group received Cb, thus lacking statistical power, second the authors’ cohort contains a small proportion of stage I patients versus stage III, this could translate into fewer pCR despite the use of Cb. Third, the authors’ criteria for TNBC subtype classification was limited to IHC/FISH instead of a molecular analysis by gene microarray. In 2011, a study by Lehmann *et al* [28] postulated the existence of six subclasses within the TNBC subtype: Immunomodulatory, Mesenchymal, Mesenchymal stem-like, Luminal androgen receptor (LAIR), Unstable and two Basal-like subclasses (BL1 and BL2). More recently, Burstein *et al* [29] performed RNA and DNA profiling analysis and defined four TNBC subclasses or clusters: cluster 1: Luminal AR (AI), cluster 2: Mesenchymal, cluster 3: Basal-like immunosuppressed and cluster 4: Basal-like immune-activated. These classifications illustrate the high heterogeneity of TNBCs that could be reflected in different responses to NACT [30]. Fourth, the authors’ study did not assess other potential biomarkers of platinum-response such as HRD, BRCA status or tumour infiltrating lymphocytes [31]. In this regard, the authors’ study found only six *BRCA1/2* mutants (3.8 %), much lower than the 20% reported in previous studies [32]. Finally, key information such as toxicity, ratio of chemotherapy delays, dose adjustments and suspension could not be obtained. Evidently, lower rates of CT completion or intensity could be a confounding factor in the lower rate of pCR. As described in the literature, reported toxicity should be critical in order to determine the true benefit of combined therapies.

## Conclusion

In summary, this work provides relevant real-world evidence that might assist in decision-making and policies to incorporate the use of available drugs into clinical practice.

## Figures and Tables

**Table 1.** Clinical characteristics of 156 Chilean TNBC patients treated with NACT, with and without platinum.

	CT without Cb	CT with Cb	Total	*p*-value
Patients; *n* (%)	136 (87)	20 (13)	156 (100)	
Average age (range)	52 (24–81)	49 (28–72)	51 (24–81)	0.34
Stage; *n* (%)^a^ I II III	4 (3) 56 (43) 71 (54)	1 (5) 11 (55) 8 (40)	5 (3) 67 (44) 79 (52)	0.48
pCR; *n* (%)	38 (28)	7 (35)	45 (29)	0.59
3-year survival (%) OS DFS DDFS	82 (74–88) 73 (64–80) 74 (65–81)	87 (55–97) 75 (41–91) 83 (48–96)	82 (75–88) 74 (65–80) 75 (66–81)	0.52 0.58 0.44

CT; Chemotherapy, Cb; Carboplatin, pCR; Pathological complete response, OS; Overall survival, DFS; Disease-free survival, DDFS; Distant disease free survival

a Five patients missing data

^b^80 % (*n* = 125) sequential anthracycline plus taxane regimen, 15% (*n* = 24) anthracyclines plus cyclophosphamides with or without fluorouracil and 4.5% (*n* = 7) only paclitaxel. One patient received cyclophosphamide methotrexate and fluorouracil

**Table 2.** Results of phase II–III clinical trials evaluating the addition of Cb to NACT schemes in TNBC. Literature review, last 10 years.

Study type	*n*	CT schedule	*n* arm	pCR% ypT0N0	*p* value	DFS HR (CI)	OS HR (CI)	Observations
**PHASE II**								
Alba* et al* [15] GEICAM/2006-03 2012	94	EC-D+Cb^a^	48	29.8	0.61	-	-	-
		EC-D	46	30.5				
Ando* et al* [21] 2014	75	TCb-FEC^b^	37	0 61a	0.003	-	-	Subgroup analysis for TN
		T-FEC	38	26				
Von Minckwitz* et al* [17] GeparSixto 2014	315	TLA Cb+B^c^	158	53^a^	0.005	0·56^a^ (0.34–0.93)	0.60 (0.32–1.12)	47 months follow up showed increase in DFS for Cb use with no differences regarding BRCA status
		TLA+B	157	37				
Sikov* et al* [17] **CALGB/ALLIANCE** 2016	218	TCb-AC^d^	111	54^a^	0.003	0.84 (0.58–1.22)	1.15 (0.74–1.79)	39 months follow up showed no survival benefits for Cb group. Improved OS for pCR
		TCb-AC TB-AC						
		T-AC	107	41				
Zhang *et al* [34] 2016	91	TCb^e^	47	38.6^a^	0.014	-	-	55 months follow up showed a 21% benefit in 5 years DFS with Cb (*p* = 0.043)
		ET	44	14				
Gluz *et al* [35] WSG-ADAPT-TN 2018	336	Nab-P+Cb^f^	154	45.9^a^	0.002	-	-	No anthracyclines used
		Nab-P+Gem	182	27.8				
PHASE III								
Loibl* et al* [20] BrighTNess 2018	318	TCb-AC^g^	160	58^a^	0.0001	-	-	No differences for BRCA status
		TCbV-AC						
		T-AC	158	31				
Schmid* et al* [27] Keynote 522 2020	1,174	TCb-AC+Pem^g^	784	65	0.0001	0.63^a^ (0.43–0.93)	-	Both arms used Cb
		TCb-AC^g^	390	51				

TN; Triple negative, CT; Chemotherapy, AUC; Area under de curve, pCR; Pathological complete response, OS, Overall survival, DFS: Disease-free survival, DDFS; Distant disease-free survival, DHR; Deficient homologous recombination, E; Epirubicin, C; Cyclophosphamide, D; Docetaxel, T; Paclitaxel, B; Bevacizumab, Cb; Carboplatin, F; Fluorouracil, Nab-P; Nab-paclitaxel, G; Gemcitabine, V; Veliparib, Pem; Pembrolizumab, A; Doxorubicin, LA; non PEGylated liposomal doxorubicin

a E 90 mg/m^2^ + C 600 mg/m^2^ q3w × 4→D 75 mg/m^2^ + Cb AUC (6 mg/mL)

b T 80 mg/m^2^ qw × 12 + Cb AUC 5 q3w × 4→ FEC: F 500 mg/m^2^ − E 100 mg/m^2^ − C 500 mg/m^2^ q3w × 4

c T 80 mg/m^2^ qw + LA 20 mg/m^2^ qw +/− Cb AUC 2 qw × 18w + B 15 mg/kg q3w × 6

d T 80 mg/m^2^ qw ×12 +/− Cb AUC6 q3w ×4→A 60 mg/m^2^ + C 600 mg/m^2^ q2w × 4

e T 175 mg/m^2^ on day 1 + Cb AUC5 on day 2 (q3w) or E 75 mg/m^2^ on day 1

f A Nab-T 125 mg/m^2^ + Cb AUC2 day 1, 8 q3w or G 1,000 mg/m^2^ dL, 8 three times weekly (q3w)

g T 80 mg/m^2^ qw ×12+/− Cb AUC6 q3w ×4 → A 60 mg/m^2^+ C 600 mg/m^2^ q2−3w ×4

^h^T 80 mg/m^2^ qw ×12 + Cb AUC6 q3w ×4 → A 60 mg/m^2^+ C 600 mg/m^2^ q2−3w ×4 +/− Pem 200 mg every 3 weeks × 8
